# Supplementary material for: Tropical volcanism enhanced the East Asian summer monsoon during the last millennium
Source: Nat Commun. 2022 Jun 14;13:3429. doi: 10.1038/s41467-022-31108-7 (PMC9197930; doi:10.1038/s41467-022-31108-7)
Supplement: Supplementary file 1 — Supplementary Information [file 41467_2022_31108_MOESM1_ESM.docx]

**Supplementary Information for “Tropical Volcanism Enhanced the East Asian Summer Monsoon during the Last Millennium”**

Fei Liu^1^, Chaochao Gao^2*^, Jing Chai^3,4^, Alan Robock^5^, Bin Wang^6,7*^, Jinbao Li^8^, Xu Zhang^9^, Gang Huang^4^ and Wenjie Dong^1^

^1^School of Atmospheric Sciences Sun Yat-Sen University, Key Laboratory of Tropical Atmosphere-Ocean System Ministry of Education, and Southern Marine Science and Engineering Guangdong Laboratory, Zhuhai 519082, China

^2^College of Environmental and Resource Sciences, Zhejiang University, Hangzhou 310058, China

^3^Plateau Atmosphere and Environment Key Laboratory of Sichuan Province, School of Atmospheric Sciences, Chengdu University of Information Technology, Chengdu 610225, China

^4^State Key Laboratory of Numerical Modeling for Atmospheric Sciences and Geophysical Fluid Dynamics, Institute of Atmospheric Physics, Chinese Academy of Sciences, Beijing 100029, China

^5^Department of Environmental Sciences, Rutgers University, New Brunswick, NJ 08901, USA.

^6^Department of Atmospheric Sciences and International Pacific Research Center, University of Hawaii at Manoa, Honolulu, HI 96822, USA

^7^Earth System Modeling Center and Climate Dynamics Research Center, Nanjing University of Information Science & Technology, Nanjing 210044, China

^8^Department of Geography, University of Hong Kong, Hong Kong SAR, China

^9^Group of Alpine Paleoecology and Human Adaptation (ALPHA), State Key Laboratory of Tibetan Plateau Earth System, Resources and Environment (TPESRE), Institute of Tibetan Plateau Research, Chinese Academy of Sciences, Beijing, China

*Nature Communications*

*To whom correspondence should be addressed. Chaochao Gao, [gaocc@zju.edu.cn](mailto:gaocc@zju.edu.cn) and Bin Wang, [wangbin@hawaii.edu](mailto:wangbin@hawaii.edu)


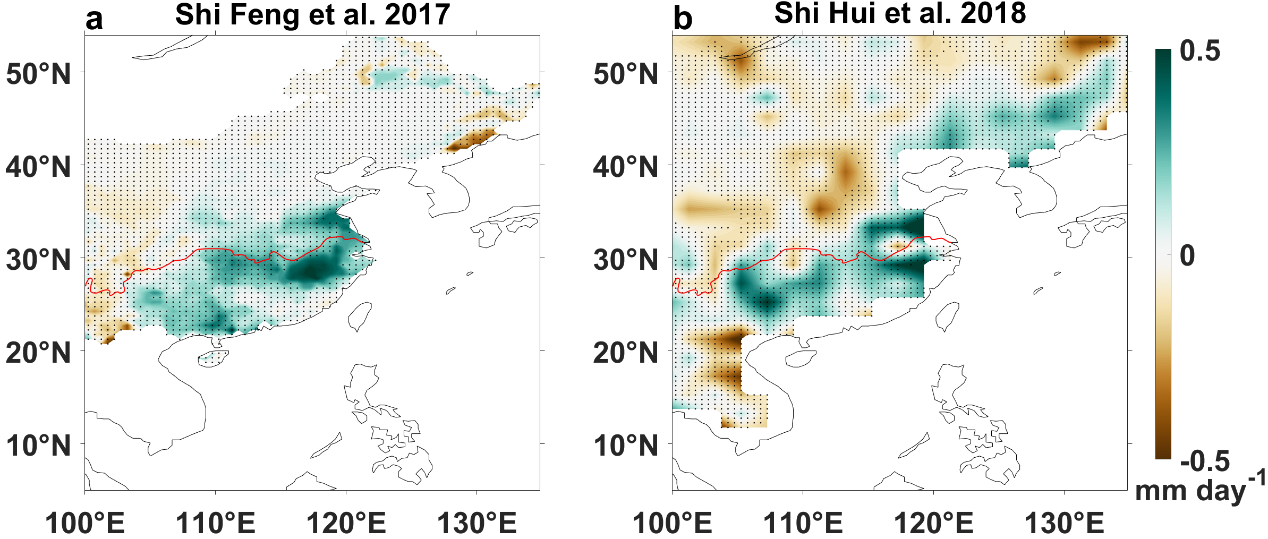


**Supplementary Figure 1. East Asian summer monsoon (EASM) response to tropical eruptions based on reconstructions of Shi et al.^1,2^.** Superposed epoch analysis results of East China precipitation anomalies (shading) in **a** Shi, et al. ^1^ and **b** Shi, et al. ^2^ in the first boreal summer after 22 tropical eruptions^3^ from 1470 to 1999 AD. Stippling indicates precipitation anomalies not significant at the 90% confidence level. The red curve is the Yangtze River. The data of Shi, et al. ^2^ were linearly interpolated to a 0.5°×0.5° grid. This figure was created using MATLAB 2020a (<http://www.mathworks.com/>).


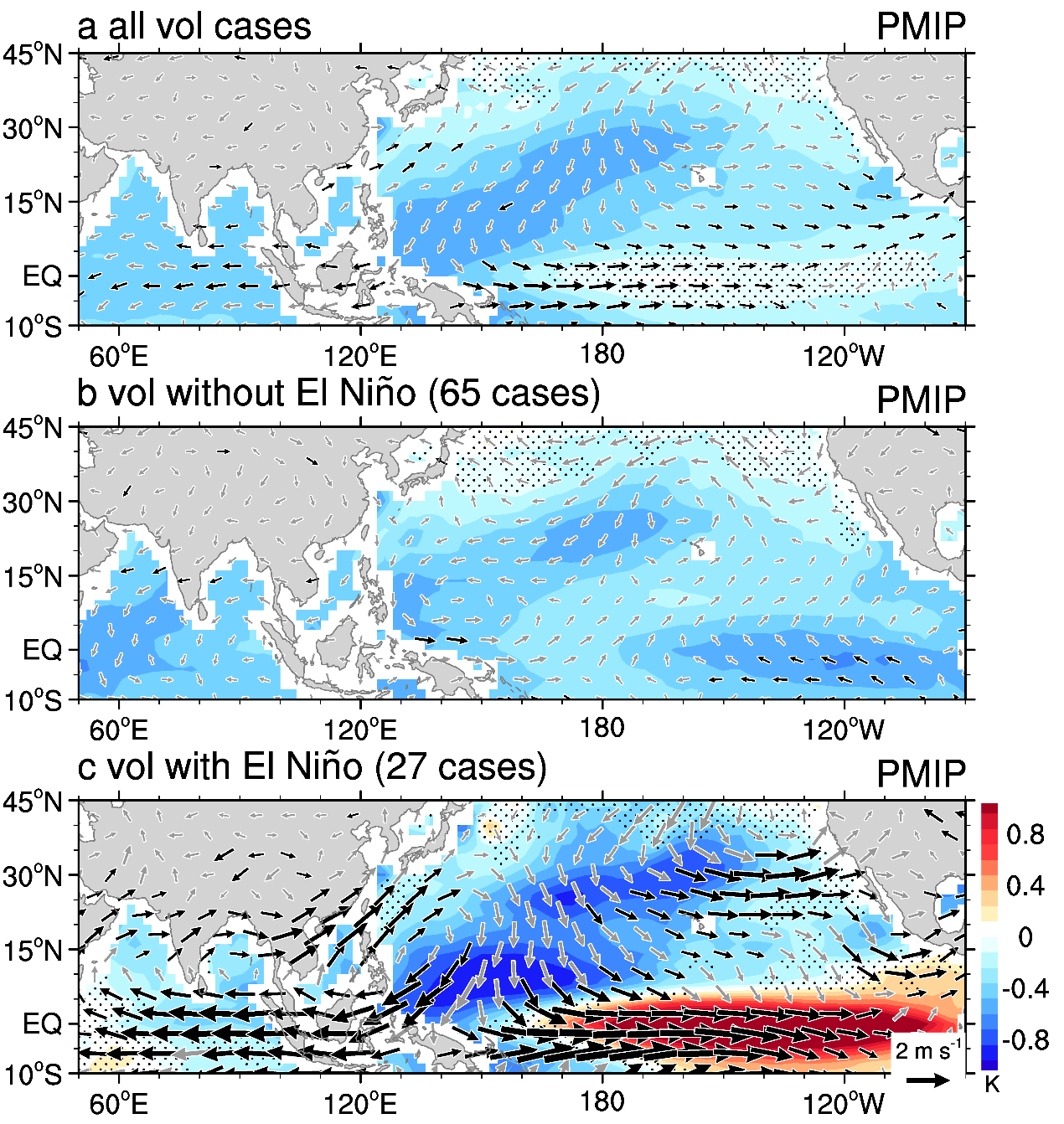


**Supplementary Figure 2. Simulated ocean responses to tropical eruptions.** Composite SST anomalies (shading) and 850 hPa wind anomalies (vectors) in the first boreal winter after **a** all 92 simulated tropical eruptions, **b** 65 eruptions without, and **c** 27 eruptions with an El Niño response in 13 PMIP last millennium simulations from 1470 to 1849 AD. Stippling and grey vectors indicate temperature anomalies and wind anomalies not significant at the 90% confidence level, respectively. Maps created with The NCAR Command Language (Version 6.6.2) [Software]. (2019). Boulder, Colorado: UCAR/NCAR/CISL/TDD. <http://dx.doi.org/10.5065/D6WD3XH5>.


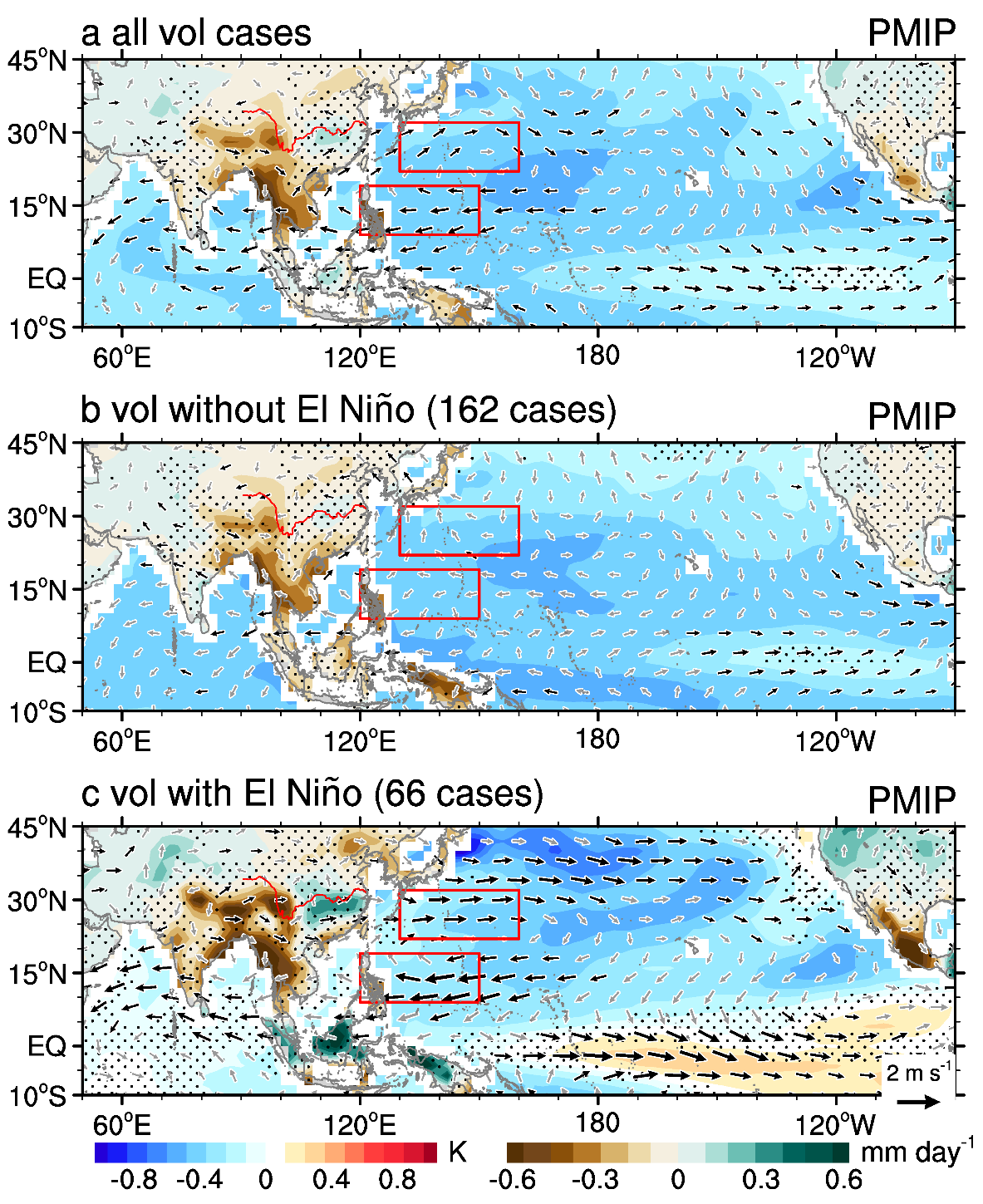


**Supplementary Figure 3. Simulated EASM-ocean responses to tropical eruptions during last millennium in PMIP models.** Composite SST anomalies (shading over ocean), precipitation anomalies (shading over land), and 850 hPa wind anomalies (vectors) in the first boreal summer after **a** all 228 simulated tropical eruptions, **b** 162 eruptions without, and **c** 66 eruptions with an El Niño response in the first boreal winter after the eruption in 13 PMIP last millennium simulations from 850 to 1849 AD. Stippling and grey vectors indicate precipitation and temperature anomalies and wind anomalies not significant at the 90% confidence level, respectively. The red curve is the Yangtze River. The red rectangles denote the locations where the EASM circulation index is defined: the 850 hPa zonal wind averaged in the southern box minus that in the northern box (see Methods). Maps created with The NCAR Command Language (Version 6.6.2) [Software]. (2019). Boulder, Colorado: UCAR/NCAR/CISL/TDD. <http://dx.doi.org/10.5065/D6WD3XH5>.


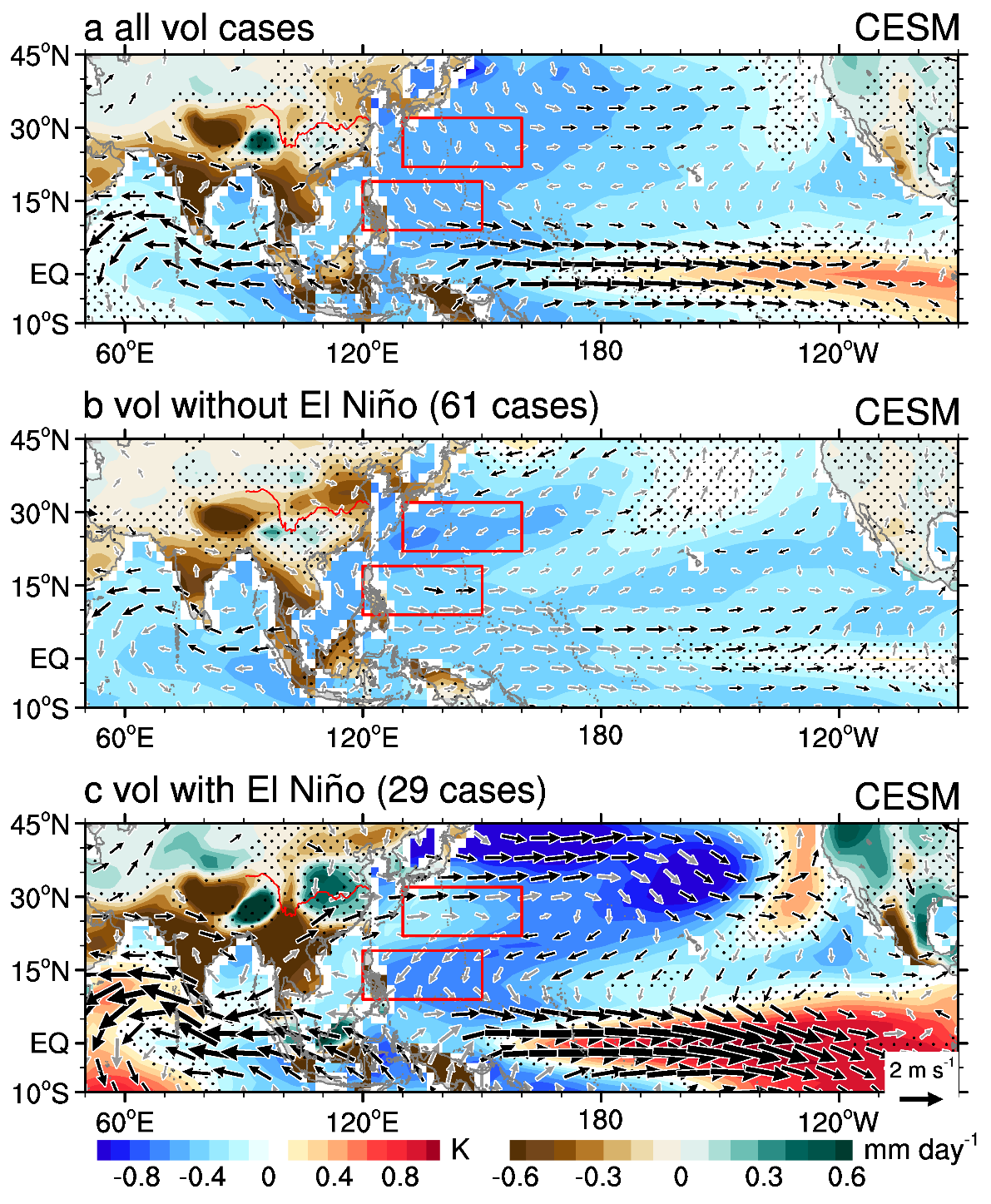


**Supplementary Figure 4. Simulated EASM-ocean responses to tropical eruptions during last millennium in CESM.** Composite SST anomalies (shading over ocean), precipitation anomalies (shading over land), and 850 hPa wind anomalies (vectors) in the first boreal summer after **a** all 90 simulated tropical eruptions, **b** 61 eruptions without, and **c** 29 eruptions with an El Niño response in the first boreal winter after the eruption in 10 full forcing ensembles of CESM from 1470 to 1999 AD. Stippling and grey vectors indicate precipitation and temperature anomalies and wind anomalies not significant at the 90% confidence level, respectively. The red curve is the Yangtze River. The red rectangles denote the locations where the EASM circulation index is defined: the 850 hPa zonal wind averaged in the southern box minus that in the northern box (see Methods). Maps created with The NCAR Command Language (Version 6.6.2) [Software]. (2019). Boulder, Colorado: UCAR/NCAR/CISL/TDD. <http://dx.doi.org/10.5065/D6WD3XH5>.

**
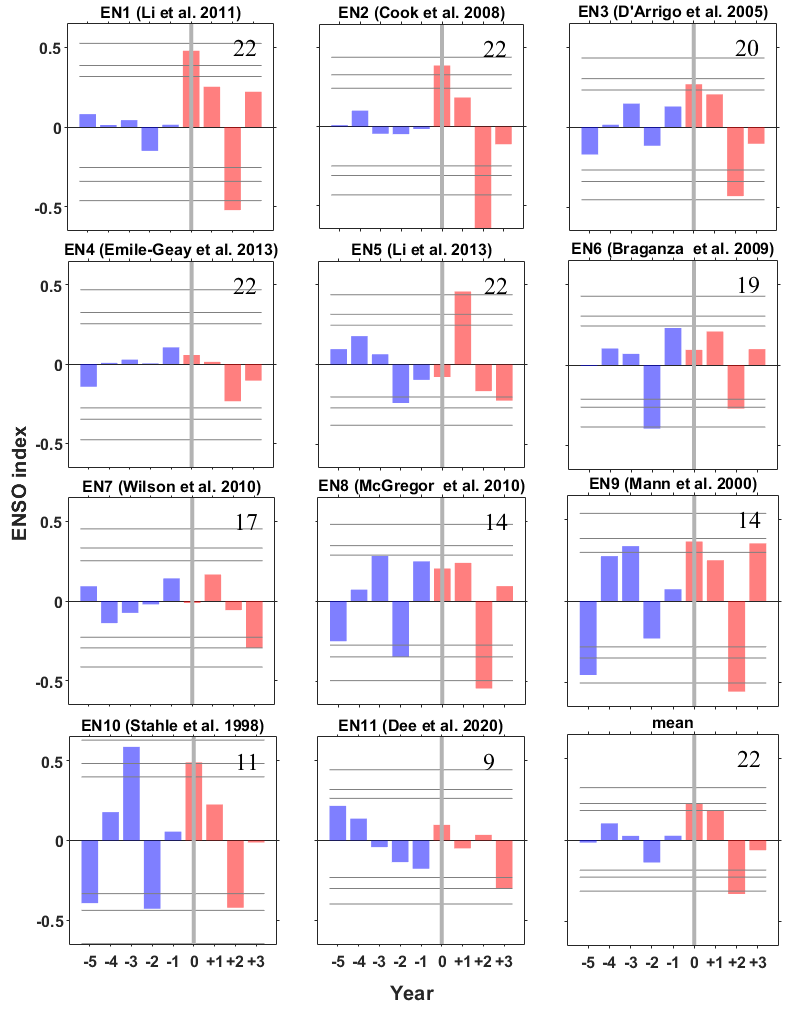
**

**Supplementary Figure 5. El Niño response to tropical eruptions based on reconstructions.** Response of individual ENSO reconstructions (Supplementary Table 1), as well as their ensemble mean, to 22 tropical eruptions (Supplementary Table 2) during 1470 to 1999 AD. Confidence limits (90, 95, 99%) are marked by horizontal lines. The ENSO index is in units of standard deviations. Blue and red colors mark the pre-eruption and post-eruption composites, respectively. “0” denotes the first cold season following the eruptions. The number of eruptions available for each ENSO reconstruction analysis is also presented.

**Supplementary Table 1.** ENSO reconstructions used in this paper.

| **Index** | **Variable/Proxy** | **Source** | **Period** |
| --- | --- | --- | --- |
| EN1 | ENSO Proxy | North American tree rings^4^ | 900–2002 |
| EN2 | ENSO Proxy | Tree rings data from Mexico and Texas^5^ | 1300–1979 |
| EN3 | ENSO Proxy | Tree rings “TEX-MEX” region North America^6^ | 1408–1978 |
| EN4 | ENSO Proxy | Network of tropical records from the circum-Pacific region^7^ | 1000–2000 |
| EN5 | ENSO Proxy | Tree rings Asia, New Zealand, North & South America^8^ | 1301–2005 |
| EN6 | ENSO Proxy | Corals, tree rings, and ice cores from the western Pacific, New Zealand, central Pacific and subtropical North America^9^ | 1525–1982 |
| EN7 | ENSO Proxy | Corals from the central Pacific, tree rings from the TexMex region and USA, and corals and ice cores from other tropical regions^10^ | 1607–1998 |
| EN8 | ENSO Proxy | Tree rings, corals and ice cores from the Pacific Basin^11^ | 1650–1977 |
| EN9 | ENSO Proxy (Niño3) | Tree rings, corals and ice cores from tropical Pacific region^12^ | 1650–2000 |
| EN10 | ENSO Proxy (SOI) | Tree rings from North America and Indonesia^13^ | 1706–1977 |
| EN11 | ENSO Proxy | Fossil corals from the central tropical Pacific^14^ | 1147–1465 1637–1705  1887–2007 |

**Supplementary Table 2.** Dates of 22 tropical volcanic eruptions (represented by the peak aerosol loading year) during 1470–1999 AD obtained from Sigl, et al. ^3^. Nine eruptions followed by an El Niño based on the ensemble mean of 11 ENSO reconstructions (see Methods) are shown in boldface.

| **Year** |
| --- |
| 1512 |
| 1554 |
| **1585** |
| 1595 |
| **1601** |
| 1641 |
| **1673** |
| 1693 |
| 1695 |
| 1762 |
| 1809 |
| **1815** |
| **1832** |
| 1836 |
| 1862 |
| **1884** |
| 1903 |
| 1943 |
| 1964 |
| **1968** |
| **1982** |
| **1991** |

**Supplementary Table 3.** Last-millennium simulations in PMIP3^15,16^ and PMIP4^17^, and in CESM-Last millennium ensembles (LME)^18^.

| **Model** | **Volcanic forcing^a^** | **Resolution**  **(lat×lon×lev)** | **Project**  **(ensembles)** | **Period**  **(A.D.)** | **References** |
| --- | --- | --- | --- | --- | --- |
| BCC-CSM-1.1 | GRA | 128×64×26 | PMIP3 (1) | 850-1850 | Wu, et al. ^19^ |
| CCSM4 | GRA | 288×192×26 | PMIP3 (1) | 850-1850 | Gent, et al. ^20^  Landrum, et al. ^21^ |
| FGOALS-s2 | GRA | 128×60×26 | PMIP3 (1) | 850-1850 | Bao, et al. ^22^ |
| MRI-CGCM3 | GRA | 320 ×160×48 | PMIP3 (1) | 850-1850 | Yukimoto, et al. ^23^ |
| IPSL-CM5A-LR | Ammann | 96×95×39 | PMIP3 (1) | 850-1850 | Dufresne, et al. ^24^ |
| CSIRO-Mk3L-1.2 | CEA | 64×56×18 | PMIP3 (1) | 851-1850 | Phipps, et al. ^25^ |
| GISS-E2-R | CEA | 144×90×40 | PMIP3 (1) | 850-1850 | Schmidt, et al. ^26^ |
| HadCM3 | CEA | 96×73×19 | PMIP3 (1) | 850-1850 | Collins, et al. ^27^  Pope, et al. ^28^  Schurer, et al. ^29^ |
| MIROC-ESM | CEA | 128×64×80 | PMIP3 (1) | 850-1849 | Watanabe, et al. ^30^ |
| MPI-ESM-P | CEA | 196×98×47 | PMIP3 (1) | 850-1849 | Jungclaus, et al. ^31^ |
| EC-Earth3-Veg-LR | Toohey | 320×160×62 | PMIP4 (1) | 850-1849 | Consortium ^32^ |
| MIROC-ES2L | Toohey | 128×64×40 | PMIP4 (1) | 850-1849 | Ohgaito, et al. ^33^ |
| MRI-ESM2-0 | Toohey | 360×160×80 | PMIP4 (1) | 850-1849 | Yukimoto, et al. ^34^ |
| CESM-LME | GRA | 144×90×26 | LME-full forcing (10) | 850-2005 | Otto-Bliesner, et al. ^18^ |

^a^GRA: Gao, et al. ^35^; Ammann: Ammann, et al. ^36^; CEA: Crowley, et al. ^37^; Toohey: Toohey and Sigl ^38^.

**Supplementary Table 4.** Dates for tropical volcanic eruptions used in PMIP3, PMIP4 and CESM during the last millennium. Eruptions during 1470-1850 AD are shown in boldface.

| **GRA^a^** | **CEA^a^** | **Toohey^a^** |
| --- | --- | --- |
| 870 (872^b^) | 971 | 1028 |
| 901 (900^b^) | 991 | 1108 |
| 961 (958^b^) | 1041 | 1171 |
| 1001 (no^b^) | 1113 | 1191 |
| 1081 (1082^b^) | 1229 | 1230 |
| 1213 | 1258 | 1257 |
| 1258 | 1286 | 1276 |
| 1275 (1278^b^) | 1331 | 1286 |
| 1284 (1286^b^) | 1456 | 1345 |
| 1341 (no^b^) | **1576** | 1453 |
| 1416 (1417^b^) | **1594** | 1458 |
| 1452 | **1674** | **1585** |
| **1600** | **1696** | **1595** |
| **1641** | **1809** | **1600** |
| **1673** | **1816** | **1641** |
| **1809** | **1835** | **1695** |
| **1815** |  | **1809** |
| **1835** |  | **1815** |
| 1883 |  | **1831** |
| 1963 |  | **1835** |
| 1991 |  |  |

^a^GRA=Gao, et al. ^35^; CEA=Crowley, et al. ^37^; Toohey=Toohey and Sigl ^38^. 1257 and 1815 is used in Toohey due to their large loading, although peaking in 1258 and 1816. ^b^The dates are chosen based on the-top-of-the-atmosphere shortwave radiation in the IPSL model that is forced by volcanic forcing of Ammann, et al. ^36^.

**Supplementary References**

1 Shi, F., Zhao, S., Guo, Z., Goosse, H. & Yin, Q. Multi-proxy reconstructions of May–September precipitation field in China over the past 500 years. *Clim. Past* **13**, 1919-1938, doi:10.5194/cp-13-1919-2017 (2017).

2 Shi, H., Wang, B., Cook, E. R., Liu, J. & Liu, F. Asian Summer Precipitation over the Past 544 Years Reconstructed by Merging Tree Rings and Historical Documentary Records. *Journal of Climate* **31**, 7845-7861, doi:10.1175/jcli-d-18-0003.1 (2018).

3 Sigl, M. *et al.* Timing and climate forcing of volcanic eruptions for the past 2,500 years. *Nature* **523**, 543-549, doi:10.1038/nature14565 (2015).

4 Li, J. *et al.* Interdecadal modulation of El Niño amplitude during the past millennium. *Nature Climate Change* **1**, 114-118, doi:10.1038/nclimate1086 (2011).

5 Cook, E., D’Arrigo, R. & Anchukaitis, K. ENSO reconstructions from long tree-ring chronologies: Unifying the differences. *Talk presented at a special workshop on Reconciling ENSO Chronologies for the Past* **500**, 15 (2008).

6 D'Arrigo, R., Cook, E. R., Wilson, R. J., Allan, R. & Mann, M. E. On the variability of ENSO over the past six centuries. *Geophysical Research Letters* **32**, doi:10.1029/2004GL022055 (2005).

7 Emile-Geay, J., Cobb, K. M., Mann, M. E. & Wittenberg, A. T. Estimating Central Equatorial Pacific SST Variability over the Past Millennium. Part I: Methodology and Validation. *Journal of Climate* **26**, 2302-2328, doi:10.1175/jcli-d-11-00510.1 (2013).

8 Li, J. *et al.* El Niño modulations over the past seven centuries. *Nature Climate Change* **3**, 822-826, doi:10.1038/nclimate1936 (2013).

9 Braganza, K., Gergis, J. L., Power, S. B., Risbey, J. S. & Fowler, A. M. A multiproxy index of the El Niño–Southern Oscillation, A.D. 1525–1982. *Journal of Geophysical Research: Atmospheres* **114**, doi:10.1029/2008JD010896 (2009).

10 Wilson, R. *et al.* Reconstructing ENSO: the influence of method, proxy data, climate forcing and teleconnections. *Journal of Quaternary Science* **25**, 62-78, doi:10.1002/jqs.1297 (2010).

11 McGregor, S., Timmermann, A. & Timm, O. A unified proxy for ENSO and PDO variability since 1650. *Climate of the Past* **6**, 1-17, doi:10.5194/cp-6-1-2010 (2010).

12 Mann, M. E. *et al.* Global temperature patterns in past centuries: An interactive presentation. *Earth interactions* **4**, 1-1, doi:10.1175/1087-3562(2000)004<0001:GTPIPC>2.3.CO;2 (2000).

13 Stahle, D. W. *et al.* Experimental Dendroclimatic Reconstruction of the Southern Oscillation. *Bulletin of the American Meteorological Society* **79**, 2137-2152, doi:10.1175/1520-0477(1998)079<2137:edrots>2.0.co;2 (1998).

14 Dee, S. G. *et al.* No consistent ENSO response to volcanic forcing over the last millennium. *Science* **367**, 1477-1481, doi:10.1126/science.aax2000 (2020).

15 Braconnot, P. *et al.* The Paleoclimate Modeling Intercomparison Project contribution to CMIP5. *CLIVAR Exchanges* **56**, 15-19 (2011).

16 Braconnot, P. *et al.* Evaluation of climate models using palaeoclimatic data. *Nature Climate Change* **2**, 417-424, doi:10.1038/nclimate1456 (2012).

17 Jungclaus, J. H. *et al.* The PMIP4 contribution to CMIP6–Part 3: The last millennium, scientific objective, and experimental design for the PMIP4 past1000 simulations. *Geoscientific Model Development* **10**, 4005-4033, doi:10.5194/gmd-10-4005-2017 (2017).

18 Otto-Bliesner, B. L. *et al.* Climate Variability and Change since 850 CE: An Ensemble Approach with the Community Earth System Model. *Bulletin of the American Meteorological Society* **97**, 735-754, doi:10.1175/bams-d-14-00233.1 (2016).

19 Wu, T. *et al.* Global carbon budgets simulated by the Beijing Climate Center Climate System Model for the last century. *Journal of Geophysical Research: Atmospheres* **118**, 4326-4347, doi:10.1002/jgrd.50320 (2013).

20 Gent, P. R. *et al.* The Community Climate System Model Version 4. *Journal of Climate* **24**, 4973-4991, doi:10.1175/2011jcli4083.1 (2011).

21 Landrum, L. *et al.* Last Millennium Climate and Its Variability in CCSM4. *Journal of Climate* **26**, 1085-1111, doi:10.1175/jcli-d-11-00326.1 (2013).

22 Bao, Q. *et al.* The Flexible Global Ocean-Atmosphere-Land system model, Spectral Version 2: FGOALS-s2. *Advances in Atmospheric Sciences* **30**, 561-576, doi:10.1007/s00376-012-2113-9 (2013).

23 Yukimoto, S. *et al.* A New Global Climate Model of the Meteorological Research Institute: MRI-CGCM3-Model Description and Basic Performance-. *Journal of the Meteorological Society of Japan. Ser. II* **90A**, 23-64, doi:10.2151/jmsj.2012-A02 (2012).

24 Dufresne, J. L. *et al.* Climate change projections using the IPSL-CM5 Earth System Model: from CMIP3 to CMIP5. *Climate Dynamics* **40**, 2123-2165, doi:10.1007/s00382-012-1636-1 (2013).

25 Phipps, S. J. *et al.* The CSIRO Mk3L climate system model version 1.0 – Part 2: Response to external forcings. *Geosci. Model Dev.* **5**, 649-682, doi:10.5194/gmd-5-649-2012 (2012).

26 Schmidt, G. A. *et al.* Present-Day Atmospheric Simulations Using GISS ModelE: Comparison to In Situ, Satellite, and Reanalysis Data. *Journal of Climate* **19**, 153-192, doi:10.1175/jcli3612.1 (2006).

27 Collins, M., Tett, S. F. B. & Cooper, C. The internal climate variability of HadCM3, a version of the Hadley Centre coupled model without flux adjustments. *Climate Dynamics* **17**, 61-81, doi:10.1007/s003820000094 (2001).

28 Pope, V. D., Gallani, M. L., Rowntree, P. R. & Stratton, R. A. The impact of new physical parametrizations in the Hadley Centre climate model: HadAM3. *Climate Dynamics* **16**, 123-146, doi:10.1007/s003820050009 (2000).

29 Schurer, A. P., Hegerl, G. C., Mann, M. E., Tett, S. F. B. & Phipps, S. J. Separating Forced from Chaotic Climate Variability over the Past Millennium. *Journal of Climate* **26**, 6954-6973, doi:10.1175/jcli-d-12-00826.1 (2013).

30 Watanabe, S. *et al.* MIROC-ESM 2010: model description and basic results of CMIP5-20c3m experiments. *Geosci. Model Dev.* **4**, 845-872, doi:10.5194/gmd-4-845-2011 (2011).

31 Jungclaus, J. H., Lohmann, K. & Zanchettin, D. Enhanced 20th-century heat transfer to the Arctic simulated in the context of climate variations over the last millennium. *Clim. Past* **10**, 2201-2213, doi:10.5194/cp-10-2201-2014 (2014).

32 Consortium, E. C.-E. EC-Earth-Consortium EC-Earth3-Veg-LR model output prepared for CMIP6 PMIP. *Earth System Grid Federation*, doi:10.22033/ESGF/CMIP6.718 (2020).

33 Ohgaito, R. *et al.* PMIP4 experiments using MIROC-ES2L Earth system model. *Geosci. Model Dev.* **14**, 1195-1217, doi:10.5194/gmd-14-1195-2021 (2021).

34 Yukimoto, S. *et al.* MRI MRI-ESM2.0 model output prepared for CMIP6 PMIP. *Earth System Grid Federation*, doi:10.22033/ESGF/CMIP6.636 (2019).

35 Gao, C., Robock, A. & Ammann, C. Volcanic forcing of climate over the past 1500 years: An improved ice core-based index for climate models. *Journal of Geophysical Research: Atmospheres* **113**, doi:10.1029/2008JD010239 (2008).

36 Ammann, C. M., Joos, F., Schimel, D. S., Otto-Bliesner, B. L. & Tomas, R. A. Solar influence on climate during the past millennium: Results from transient simulations with the NCAR Climate System Model. *Proceedings of the National Academy of Sciences* **104**, 3713-3718, doi:10.1073/pnas.0605064103 (2007).

37 Crowley, T. J. *et al.* Volcanism and the little ice age. *PAGES news* **16**, 22-23, doi:10.1029/2002GL0166335 (2008).

38 Toohey, M. & Sigl, M. Volcanic stratospheric sulfur injections and aerosol optical depth from 500 BCE to 1900 CE. *Earth Syst. Sci. Data* **9**, 809-831, doi:10.5194/essd-9-809-2017 (2017).
